# Non-Coding RNAs Regulating Mitochondrial Functions and the Oxidative Stress Response as Putative Targets against Age-Related Macular Degeneration (AMD)

**DOI:** 10.3390/ijms24032636

**Published:** 2023-01-30

**Authors:** Juha M. T. Hyttinen, Janusz Blasiak, Kai Kaarniranta

**Affiliations:** 1 Department of Ophthalmology, Institute of Clinical Medicine, University of Eastern Finland, P.O. Box 1627, FI-70211 Kuopio, Finland; 2 Department of Molecular Genetics, University of Lodz, Pomorska 141/143, 90-236 Lodz, Poland; 3 Department of Ophthalmology, Kuopio University Hospital, P.O. Box 100, FI-70029 Kuopio, Finland

**Keywords:** age-related macular degeneration, epigenetic therapy, mitochondria, non-coding RNA, oxidative stress

## Abstract

Age-related macular degeneration (AMD) is an ever-increasing, insidious disease which reduces the quality of life of millions of elderly people around the world. AMD is characterised by damage to the retinal pigment epithelium (RPE) in the macula region of the retina. The origins of this multi-factorial disease are complex and still not fully understood. Oxidative stress and mitochondrial imbalance in the RPE are believed to be important factors in the development of AMD. In this review, the regulation of the mitochondrial function and antioxidant stress response by non-coding RNAs (ncRNAs), newly emerged epigenetic factors, is discussed. These molecules include microRNAs, long non-coding RNAs, and circular non-coding RNAs. They act mainly as mRNA suppressors, controllers of other ncRNAs, or by interacting with proteins. We include here examples of these RNA molecules which affect various mitochondrial processes and antioxidant signaling of the cell. As a future prospect, the possibility to manipulate these ncRNAs to strengthen mitochondrial and antioxidant response functions is discussed. Non-coding RNAs could be used as potential diagnostic markers for AMD, and in the future, also as therapeutic targets, either by suppressing or increasing their expression. In addition to AMD, it is possible that non-coding RNAs could be regulators in other oxidative stress-related degenerative diseases.

## 1. Introduction

Oxidative stress and mitochondrial dysfunction are important factors not only in the normal ageing process but also in the development of age-related degenerative diseases such as Parkinson’s (PD) and Alzheimer’s diseases (AD), and age-related macular degeneration (AMD) of the eye [1,2]. In recent years, a large number of epigenetic factors, i.e., non-coding RNAs, have been discovered within cells, e.g., microRNAs (miRNAs), long non-coding RNAs (lncRNAs), and circular RNAs (circRNAs), where they control many cellular processes. Many of these non-coding RNAs have been demonstrated to exert an influence on the levels of oxidative stress, mitochondrial homeostasis, and the antioxidant response, and thus disturbances in their expressions might be involved in the pathology of many diseases, including AMD [3,4,5,6].

We present here a number of non-coding RNAs which disturb or enhance mitochondrial functions, or alternatively, affect the oxidative stress response, although it should be emphasized that many of them have not been proven to be directly involved in AMD pathology. Nonetheless, since it is known that both disorders in mitochondrial function and the inadequate control of the oxidative stress play crucial roles in AMD pathology, we believe that disturbances in the expressions of non-coding RNAs are topics that should be investigated in the hope of finding novel approaches to treat this devastating eye disease [2].

## 2. AMD—General

Although AMD is a rapidly increasing problem in countries all around the world, today it is the main reason for legal blindness and sight loss in western countries. While it represents a severe physical and mental problem for the affected individuals and a serious burden for societies, its pathogenesis is still poorly understood, a fact that limits therapeutic options. It is currently a major, but largely untreatable, disease.

**Figure 1:**
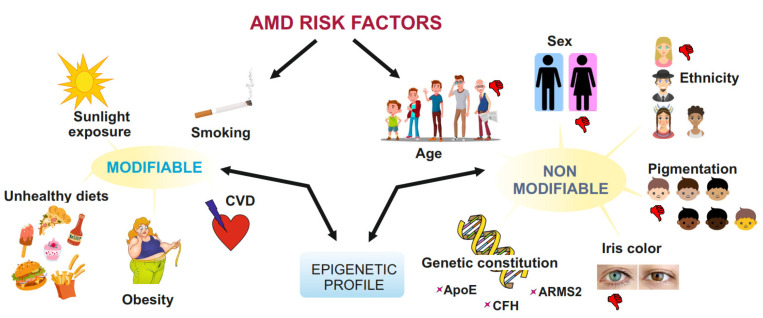
Risk factors for age-related macular degeneration. They can be divided into non-modifiable and modifiable factors. Ageing is per definition the most serious AMD risk factor, and smoking is the most consistently reported modifiable risk factor. Mutations in the genes encoding regulators of complement H activity (*CFH*), apolipoprotein E (*apoE*) and age-related maculopathy susceptibility 2 (ARMS2) as well as collagen synthesis, lipid metabolism/cholesterol transport, receptor-mediated endocytosis, endodermal cell differentiation, angiogenesis, and extracellular matrix organisation have been reported to be associated with AMD in as many as 65% of cases. Other factors, such as European/North American ethnicity, white skin pigmentation, light iris color, unhealthy diet, cardiovascular diseases and obesity, and blue light originating mostly from sunlight are less well documented and more controversial, but it cannot be excluded that a combination of these factors may significantly increase AMD risk. Both modifiable and non-modifiable AMD risk factors are mutually dependent on the epigenetic regulation of gene expression, and this is mainly determined by the cellular epigenetic profile.

The main risk factors for AMD include ageing, smoking, physical inactivity, obesity, diets high in trans- and unsaturated fats, hypercholesterolemia, hypertension, and mutations in some AMD-susceptibility loci [7] (Figure 1). Several molecular processes have been proposed to play a role in the development of AMD and many of them have been linked to oxidative stress [8].

**Figure 2:**
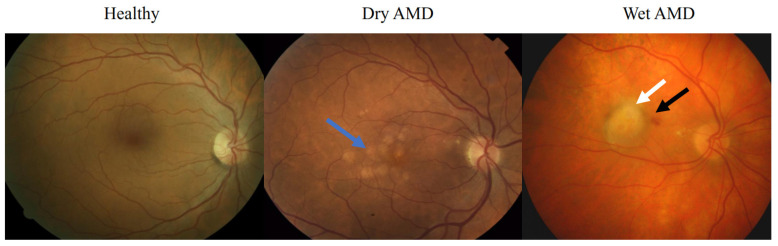
Fundus photographs from the macula area. Drusen is indicated by a blue arrow, retinal oedema with a white arrow, and a haemorrhage with a black arrow.

AMD occurs in two main forms: dry AMD (dAMD) and wet AMD (wAMD). Dry AMD causes a slowly progressing sight impairment, and comprises about 85–90% of all AMD cases [9]. It is manifested as visual distortions, including the waving of straight lines. In ageing, intracellular lipofuscin deposits start appearing in the quiescent RPE cells and this process is potentiated by the presence of other AMD risk factors [10]. These lipofuscin structures contain mainly oxidized proteins and lipids [11]. In addition to lipofuscin, drusen deposits (Figure 2) start to accumulate outside the RPE. These are yellow-white coloured extracellular debris which contain largely cholesterol, complement proteins, apolipoproteins, and carbohydrates, and the expansion of drusen leads to a gradual loss of sight [12,13]. It is interesting that drusen contains the amyloid-β oligomer; deposits of this molecule in the brain are a molecular hallmark of AD [14]. In wAMD, choriocapillaris vessels sprout into the sub- and intraretinal zones [15], but since these vessels are fragile, they often lead to haemorrhages into the retina (Figure 2). These symptoms appear suddenly and worsen quickly, leading to permanent visual loss if not properly treated [16].

Today, there is no cure for dAMD, whereas for wAMD, anti-vascular endothelial growth factor inhibitors (anti-VEGF) intravitreal injections are routinely used in the clinic [13]. However, anti-VEGF drugs only suppress choroidal neovascularisation (CNV) activity and do not stop the pathological degenerative process. Therefore, at present, the aim is to find a means to reduce the deterioration of AMD-related vision by identifying novel targets and therapies that could stop or at least slow down the progression of AMD.

## 3. Oxidative Stress and Mitochondrial Function in AMD

The term oxidative stress refers to a disturbance in cellular redox homeostasis which results from an imbalance between oxidation levels and the antioxidant defense mechanisms. Oxidative stress can be induced by various physical, chemical, and biological factors; it leads to the production of reactive oxygen species (ROS), such as peroxides, hydroxyl radical, hydrogen superoxide, and singlet oxygen. Under normal circumstances, ROS can act as effector and signaling molecules, but when produced in excess or inappropriately localized, they can damage cellular macromolecules. Endogenous ROS are produced mainly by plasma membrane-located NADPH oxidases (NOXs), and by mitochondria where they are produced as a byproduct of oxidative phosphorylation (OXPHOS), and therefore, their production increases when this process is impaired. This results in mitochondrial DNA (mtDNA) damage, oxidation of mitochondrial proteins and lipids and ultimately in a mitochondrial dysfunction. As mitochondria are the main energy source in the cell, damage to these organelles triggers deleterious changes in the cell and organism and thus forms the basis for the development of many pathological states [17,18,19]. Mitochondria are vital organelles since they provide energy for the cell by OXPHOS and adenosine triphosphate (ATP) production, which takes place through the transfer of electrons between complexes I-IV in the electron transport chain (ETC). In addition, mitochondria may play a role in other effects associated with oxidative stress, such as protein clearance and cell death [2]. Mitochondria are integrated in a communicative network, both with other mitochondria and other cellular compartments.

Mitochondrial dysfunction has been associated with ageing and many diseases commonly encountered in the elderly, including AMD [2]. For example, the presence of small-sized mitochondria has been observed in the RPEs of aged individuals [20]. In AMD subjects, mitochondrial dysfunction, damage to mtDNA and deficiencies in its repair, increases in ROS production and protein aggregation, weakened autophagy, and augmented inflammation are traits that have been observed [2,21,22]. A subtype of autophagy, mitophagy, is important in the removal of nonfunctional and redundant mitochondria; its impairment has been found play a role in RPE dysfunction and is thus speculated to represent one factor in the progression of AMD [22,23]. These findings all support the importance of the mitochondria in the RPE in the pathogenesis of AMD and their potential as a therapeutic target.

Transcription coactivator peroxisome proliferator-activated receptor gamma coactivator-1 alpha (PGC-1α), encoded by the *PPARGC1A* gene in humans, is a central regulator of cellular energy metabolism. This factor stimulates mitochondrial biogenesis, energy control and promotes the antioxidant response [24]. PGC-1α is expressed abundantly in tissues with a high number of mitochondria and active oxidative metabolism, such as RPE [25]. It is activated by ROS, stress, cold, caloric restriction, and cytokines. It has been reported, that PGC-1α regulates autophagy, mtDNA replication and stability, and estrogen-related receptors; it also activates nuclear respiratory factors and cAMP response element binding protein (CREB), which is another transcription factor, and thus stimulates the cytochrome c oxidase subunit (COX) and eventually OXPHOS [25,26]. It was demonstrated in a PGC-1α knockout mouse model that dysregulated mitochondria evoked RPE damage and visual loss [27]. In addition, disorganisation and loss of epithelial integrity have been observed [28,29]. It has been reported that the knockout of the mouse *ppargc1a* gene induces an endothelial-to-mesenchymal transition (EMT). EMT is a cellular trans-differentiation process in which cells lose their endothelial properties which are maintained by cell junctions, and gain mesenchymal, motile features. An elevation in the activity of PCG-1α promotes RPE metabolism and confers protection against ROS [30]. Furthermore, EMT was triggered in PTEN kinase 1 (PINK1)-deficient mice, manifesting as impaired mitophagy and disturbed mitochondrial function [23]. In another recent study, EMT induction by exposure to transforming growth factor beta 2 in an RPE cell culture was found to cause mitochondrial dysfunction, evident as a reduced OXPHOS and downregulation of the genes which control mitochondrial dynamics [31]. In these cells, a stimulation of PGC-1α minimized the oxidative stress damage, which suggests that there could be a relationship between energy metabolism and anti-oxidants in RPE. With respect to AMD pathology, this is certainly an important finding, and therefore, the possible linkage between PCG-1α activity and AMD pathogenesis should be confirmed [28,32].

PGC-1α functions as a co-regulator of the protein complex of Kelch-like ECH-associated protein 1 (KEAP1) and nuclear factor erythroid 2-related factor 2 (NFE2L2; NFE2 like bZIP transcription factor 2), which functions in the antioxidant response signaling [33]. Under normal conditions, KEAP1 and the Cullin3 ubiquitin ligase complex bind to the transcription co-factor NFE2L2, keeping it in the cytosol. Finally, Cullin 3 ubiquitinates NFE2L2 and leads to the proteasomal degradation of this factor. In conditions of oxidative stress, critical cysteine residues are targeted in KEAP1, and then NFE2L2 departs from the complex, allowing it to be transferred to the nucleus, where it activates the expression of various antioxidant genes by binding to the antioxidant response elements (AREs) in their promoters [34]. It has been speculated that a failure of KEAP1/NFE2L2 coordination to sense oxidative stress might have a role in the development of AMD [34]. It has also been reported that a knock-out of the *NFE2L2* gene caused RPE degeneration, accumulations of lipofuscin and drusen, as well as an increase in the level of inflammation, and increased accumulation of autophagic bodies [35]. The double knockout of genes coding PGC-1α and NFE2L2 in mice triggered increased autophagy, reduced proteasomal cleansing, mitochondrial damage, RPE degeneration, and finally the loss of vision in the animals [27]. Taken together, the weakened response against oxidative stress in RPE and the consequential mitochondrial damage may well have a role in the development of AMD. The manipulation of the antioxidant response and the restoration of correct mitochondrial function might represent a new approach for the therapy of this devastating ocular disease.

## 4. Non-Coding RNAs

### 4.1. Overview

Only a small proportion of the human genome encodes for polypeptides/proteins, although these genes are transcribed to a much greater extent [36]. The human transcriptome can be understood in at least two ways: as the total RNA content of the cell or the complete set of mRNAs. In general, the RNA content of the cell can be divided into coding (mRNA, about 4% of the total RNA) and non-coding (ncRNA, the remaining 96%) RNAs. The latter can be further divided into housekeeping and regulatory ncRNAs. The former group comprises ribosomal RNAs (rRNAs) and transfer-RNAs (tRNAs). Regulatory ncRNAs include short non-coding RNA (snRNA, fewer than 200 nucleotides in length) and long non-coding RNA (more than 200 nucleotides). It is not the intent of this review to provide detailed information on classification and general properties of non-coding RNAs as that can be found elsewhere [37,38]. We will limit our considerations here to microRNAs (miRNAs), long non-coding RNAs (lncRNAs), and circular non-coding RNAs (circRNAs).

Recent research has clarified the importance of these RNAs and demonstrated that they are important regulators of proteins and their genes. MicroRNAs are the best characterized group of these RNAs, but recent data focusing on the longer ncRNAs has revealed their importance. Epigenetic control by ncRNAs is a major interest of researchers, not only due to its significance in the control of gene expression, but also for its therapeutic potential. Many ncRNAs have been linked to various cancers and other disorders, including neurodegenerative diseases. However, their clinical applications are still some way from becoming a reality [38,39,40]. There are some recent reviews evaluating the potential of ncRNAs, mostly miRNAs in the diagnosis and therapy of AMD [3,4,41]. In the present work, we will focus on the ncRNAs involved in the regulation of mitochondria-related oxidative stress and the antioxidant response, issues which are important in the pathogenesis of AMD.

### 4.2. Micro-RNAs

MicroRNAs (miRNAs) are 20–22 bases long, regulatory RNAs, derived from their longer RNA precursors. Mammalian miRNAs are capable of regulating most genes. In humans, it has been estimated that there may be at least 2600 mature miRNAs [42,43]. In addition, each single miRNA species can target perhaps in excess of 500 transcripts [44]. Therefore, these miRNAs must be viewed as crucial factors in the epigenetic control of the largest part of cellular functions.

Mechanistically, miRNAs bind to complementary sequences in mRNAs; usually the target sites are located in the 3′-untranslated regions (3′-UTRs) in mRNA. The end result is the silencing of a gene, either by cleavage of mRNA in its binding site, destabilisation of the mRNA by shortening of the poly(A) tail, or impeding the translation of mRNA [45]. The requirement for a perfect complementarity of a miRNA with its target mRNA is only about 8 nt. This selectivity-determining region is called the seed sequence and is located in the 5′-region of miRNA. The presence of a supplementary binding region in miRNA might compensate for some mismatches in the seed region binding [39].

**Figure 3:**
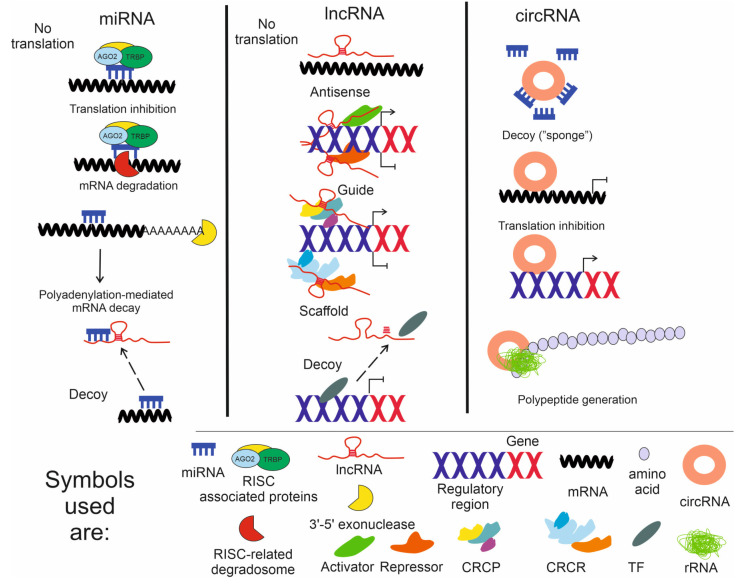
ncRNAs. Basic modes of the cellular action of microRNA (miRNA), long non-coding RNA (lncRNA) and circular RNA (circRNA) in the regulation of gene expression. After biogenesis and procession, single-stranded miRNA may bind the RNA interference silencing complex (RISC)-associated proteins containing Argonaute 2 (AGO2), the TARBP2 subunit of the RISC loading complex (TRBP) and others; when the sequence of miRNA is identical to the sequence in an mRNA, hybridisation occurs, and this double-stranded DNA blocks translation. In the case when miRNA is only partly complementary to mRNA, a degradosome, the nature of which is not completely clear, is recruited and leads to the degradation of this mRNA. miRNA may cause destabilisation and a subsequent decay of mRNA by recruiting a 3′-5′ exonuclease which degrades the poly(A) tail present at the 3′ termini of most mammalian mRNAs. The poly(A) tail is not presented in the other RNAs for the sake of simplicity. Four basic modes of lncRNAs action are described: antisense, guide, scaffold and decoy. LncRNA may pair with a complementary fragment of mRNA, preventing or inhibiting its translation, and in addition, it recruits and/or guides transcriptional activators and repressors to activate/repress transcription of the target gene. LncRNA may serve as a platform (scaffold) to facilitate the assembly of a chromatin remodeling complex to change the structure of chromatin into a more open (CRCP-chromatin remodeling complex acting permissively) or closed (CRCR-chromatin remodeling complex acting repressively) configuration. Furthermore, lncRNA may act as a decoy to recruit (broken arrows) miRNAs or transcription factors (TFs) and sequester them so that they do not bind to their target mRNA or DNA, respectively. Only examples of the properties of lncRNAs in gene expression regulation are presented, and many other mechanisms, e.g., those related to translation and post-translational regulations, are not illustrated, but in general, they follow the presented schemes.

In brief, the generation of a miRNA begins with the transcription of its gene by RNA polymerase II, producing a primary microRNA (pri-miRNA). MiRNA genes are typically located in introns of protein-coding genes or in the intergenic regions. The nuclear pri-miRNA, which forms a hairpin structure, is processed by the Drosha endonuclease to about a 60-nt long precursor, called a pre-miRNA, with a hairpin end. The pre-miRNA is transported by the exportin 5-system from the nucleus to the cytoplasm, where it is processed by RNase II DICER1 into 20–22-nt long double-stranded miRNA and its one (driver) strand binds to the RNA interference silencing complex (RISC)-associated proteins containing Argonaute 2 (AGO2), the TARBP2 subunit of the RISC loading complex (TRBP) and others, whereas the other (passenger) strand is degraded. The driver strand and its seed sequence are exposed, and finally bind to the target mRNA (Figure 3, miRNA), which leads to a degradation of the mRNA and thus inhibition of translation. In addition, miRNAs may regulate genes encoding proteins involved in chromatin remodeling, so they can be indirectly involved in transcription modulation. An RNase DICER1 deficiency in mice has been shown to promote atrophy and neovascular pathology in the retina [46]. In addition, this deficiency is manifested in dAMD eyes as an accumulation of *Alu* transcripts, originating from the most common transposable DNA elements in the human genome [47]. Furthermore, an enrichment of the *Alu* transcript in dAMD eyes has been reported to be followed by the escape of mitochondrial DNA and the activation of inflammasomes [48].

### 4.3. Long Non-Coding RNAs

While they were initially thought to have no biological function, the development of bioinformatics and deep sequencing has revealed that long non-coding RNAs (lncRNAs) are abundant, conserved, and have diverse functions in mammalian cells. LncRNAs form a diverse class of transcripts that do not serve as templates for protein synthesis. Their length is 200 nucleotides (nt) or more; in fact, they can be as long as hundreds of thousands of nucleotides. The total number of different lncRNAs in humans is still an open question, and estimates vary considerably, ranging between 16,000 and 90,000 [49].

LncRNAs are evolutionarily less well conserved, their coding genomic sequences contain fewer exons, and they are expressed at much lower levels compared with protein-coding genes. They can act as signaling molecules, regulate chromatin structure and its repair, serve as molecular guides or scaffolds, and function as sponges for other RNAs and proteins [50,51]. The regulation of chromatin structure by lncRNAs points to their involvement in the regulation of gene expression at the transcriptional level. These kinds of lncRNAs facilitate the assembly of a chromatin remodeling complex, acting on transcription either permissively or repressively (Figure 3, lncRNA). LncRNAs contain two types of functional parts: the interacting elements direct the physical contacts with other RNAs and proteins, and their structural elements determine the assembly of secondary and tertiary structures, which spatially guide their functional interactions. This means that lncRNAs have more flexible ways to interact with other nucleic acids and proteins than miRNAs. LncRNAs can modulate the binding of transcription factors and influence the stability or the translation efficiency of mRNAs. Furthermore, the interaction between lncRNAs and a protein may influence its activity and/or localisation [52]. In summary, they can modulate cellular function by regulating the transcription of other genes and by controlling the functions and localisations of proteins. In addition, lncRNAs can display unique expression patterns depending on the developmental stage or on tissue or cell type specific features [53].

Most lncRNAs are produced by the canonical, RNA polymerase II-led transcription machinery in the nucleus. Like mRNAs, they can be modified post-transcriptionally by capping, polyadenylation, and splicing. Their origin can be intergenic, as well as intronic, or even arise from the exon of protein coding genes. In addition, they can be transcribed from either the sense or antisense strand of a gene. A greater proportion of lncRNAs than miRNAs is localized in the nucleus, a phenomenon called nuclear retention [54]. This can be a result of their tethering to the chromatin, inefficient splicing due to weak splicing signals, or the presence of splicing inhibitors. However, some lncRNAs are exported via the nuclear RNA export factor 1 (NXF1) pathway to the cytosol where lncRNAs can become bound to RNA-binding proteins, bind with ribosomes, be transported into mitochondria by so far unknown mechanisms, or find their ways to reach other organelles, such as exosomes [38,55].

### 4.4. Circular Non-Coding RNAs

CircRNAs are single-stranded RNAs which form a covalently closed loop. As they have no 5′- and 3′-ends, they are more resistant to RNA exonucleases, and thus, are more stable than linear RNAs. Their size ranges from approx. 100 nt to over 1000 nt, being on average about 700 nt-long [56,57]. According to one recent estimate, over 30,000 different circRNAs may be present in humans [58]. With regard to their lengths, circRNAs can be considered as a subtype of the long non-coding RNAs.

CircRNAs were initially thought to be simply side products from the splicing of precursor-mRNAs (pre-mRNAs), thus originating from discarded intron sequences, and perhaps possessing only minimal cellular functionality. However, it has been discovered that circRNAs are formed by non-canonical splicing from pre-mRNA exons in a process called “head-to-tail” joining or backsplicing. It has even been suggested that the formation of circRNAs can be a competing event to the canonical mRNA splicing [59]. Briefly, pre-mRNAs are synthesised by RNA polymerase II and then are processed to produce a mature transcript via splicing that joins exons and removes introns. circRNAs can be formed in the process of backsplicing of exons. When the action of the spliceosome is inhibited or its components deleted, the newly-synthesised pre-mRNA strand can be redirected to alternative pathways which facilitate the backsplicing event and thus lead to the generation of circular RNA. In addition, circRNAs can retain intronic sequences, which are located between back-spliced exons [59,60]. A small portion of circRNAs contain exclusively intronic sequences which are derived from primary RNA transcript splicing waste and are called circular lariat RNA [61]. As a particularity in humans, the *Alu* repeats present in introns have been reported to mediate backsplicing [57].

Circular ncRNAs, can act as sponges, scaffolds, decoys, and recruiters for other RNAs and RNA-binding proteins (Figure 3, ncRNA). Although a large number of circRNAs have been identified, many aspects of their biology, such as their expression, transport, degradation, and function, are still something of a mystery [62]. For example, the regulation of the biogenesis of circRNA has still to be clarified [63]. In addition, circRNAs can be translated into proteins, thus adding to their already complex role in metabolic control [64].

## 5. Non-Coding RNAs in Mitochondrial Regulation

### 5.1. MiRNAs as Bioenergy Regulators in the Mitochondria

Selected miRNAs involved in the control of mitochondrial functions and oxidative stress will now be discussed (Table 1). It should be emphasized that while a fraction of them have been demonstrated to have a direct link to AMD, it is possible that they have the ability to modulate pathways important in the pathogenesis of this disease. Our selection is based firstly on if they are related to AMD, and then additional examples of miRNAs are selected from the recent literature. This applies to the lncRNAs and circRNAs discussed below in Section 5 and Section 6.

Several miRNAs target the cytochrome c oxidase (COX) subunits. For example, miR-26a and miR-26b were demonstrated to downregulate COX5a in myocardial cells in hypoxic conditions in rat myoblast cells and in rats in vivo [65], whereas miR-181c was able to bind to the 3′-UTR of the *COX1* gene, a catalytic element of complex IV of the ETC [66,67]. miR-181c was upregulated in NFE2L2-silenced human carcinoma cells, and this has been linked to the decrease in COX1 [68]. This might be of interest regarding AMD since the retina is exposed to increased hypoxia and declines in the activity of NFE2L2 parallel the signs of this disease [27,35].

In rat cardiomyocytes, miR-210 targeted COX10, another component of respiratory complex IV, and suppressed iron-sulfur cluster assembly enzyme (ISCU) gene expression in hypoxic conditions [69]. This miRNA also decreased heme levels [70]. The retinal expression of miR-210 has been reported to be upregulated in the pathological neovascularisation which occurs in response to hypoxic conditions [71].

**Table 1:** Selected microRNAs affecting mitochondrial and oxidative balance in the cell (listed in numerical order). The abbreviations are found in the corresponding text. ^a^ Indirect actions. Arrows (↑ and ↓) indicate up- or down-regulation, respectively.

miRNAs	Targets	Effect	Model	References
1 ↑	*MINOS*, *GPD2*, *LRPPRC*	Mitochondrial damage ↑, mitophagy ↑	Human breast cancer and melanoma cells	[72]
7 ↑	*KEAP1*	Antioxidant response ↑	Human neuroblastoma cells	[73]
9 ↑	^a^ PGC-1α ↑	Mitochondrial function ↑	Human kidney cells	[74]
15b, 16, 95 ↑	*Arl2*	ATP production ↓	Rat cardiomyocytes	[75]
17, 18a, 19a/b, 20a, 92 ↑	*MFN1*	Mitochondrial fusion ↓	Human osteosarcoma cells	[76]
19b-3p, 221-3p, 222-3p ↑	*PPARGC1A*	Mitochondrial function ↓	Human atherosclerotic vessel	[77]
23a ↑	*GLS1* *MnSOD*	Glutamine metabolism ↓ Mitochondrial function ↓	Human RPE cells Mouse cardiomyocytes	[78,79]
23a-3p ↑	*PPARGC1A*	Mitochondrial function and fatty acid metabolism ↓	Mouse liver	[80]
24-3p ↑	*KEAP1*	Antioxidant response ↑	Mouse cardiomyocytes	[81]
26a/b ↑	*COX5a*	OXPHOS ↓	Rat myoblasts, rat	[65]
27a ↑	*NFE2L2*	Antioxidant response ↓	Human and rat kidney cells	[82]
27a/b ↑	*PINK1*	Oxidative stress ↑	Human cervical cancer and neuroblastoma cells	[83]
29a/b, 124 ↑	*MCT1*	Pyruvate circulation ↓	Human and mouse pancreatic cells	[84,85]
33a/b ↑	*CROT*	Fatty acid oxidation ↓	Monkey liver cells	[86]
34a ↑	*NFE2L2* *PINK1*	Antioxidant response ↓ Mitophagy ↓	Neuroblastoma cells Human kidney cells, mouse	[87,88]
34b/c ↓	^a^ Parkin ↓, DJ-1 ↓	Mitochondrial function ↓	Parkinson’s disease human tissue	[89]
98 ↓	*Hey2* (Notch signaling)	Oxidative stress ↑, mitochondrial function ↓, apoptosis ↓, and viability ↓	Alzheimer’s disease mouse model	[90]
101 ↑	*PRDM16*	Mitochondrial function ↓, apoptosis ↑	Human astrocytoma cells, in silico	[91]
130-3p ↑	*PPARGC1A*	Mitochondrial function ↓, TFAM ↓	Human placental cells	[92]
142, 144, 153 ↑	*NFE2L2*	Antioxidant response ↓	Human neuroblastoma cells	[93]
181a ↑	*PARKIN*	Mitophagy ↓	Human neuroblastoma cells	[94]
181a/b ↑	*NRF1*, *COX11*, *COQ10B*, *PRDX3*	Mitochondrial biogenesis and function ↓	Mouse retinal neurons	[95]
181c ↑	*COX1*	OXPHOS ↓	Rat myocytes	[66,67]
204 ↑	*PPARGC1A*	Mitochondrial copy number ↓, citrate cycle function ↓, autophagy ↓	Mouse myoblast cells	[96]
210 ↑	*COX10*	OXPHOS ↓	Human primary fibroblasts	[69]
210 ↑	*Ephrin-A3*	Tubulogenesis and chemotaxis ↑	Human umbilical vein and osteosarcoma cells	[97]
338 ↑	*COX4, ATP5G1*	OXPHOS ↓	Primary rat neuronal cells	[98,99]
494 ↑	*PARK7*	Antioxidant response ↓	Mouse adipocyte and neuroblastoma cells	[100]
762 ↑	*ND2*	OXPHOS ↓	Mouse cardiomyocytes	[101]

This might be due to the down-regulation of tyrosine kinase ligand ephrin-A3, which is a direct target of miR-210, since there is a report that the down-regulation stimulates the tubulogenesis and chemotaxis that occurs in human umbilical vein and osteosarcoma cells under hypoxic conditions [97]. Thus, the miR-210/ephrin-A3 pathway might well be linked to the progression of AMD.

MiR-338 was found to modulate the expressions of the mitochondrial COX4 protein and ATP synthase ATP5G1, a key component in the complex V of the OXPHOS chain. If its levels were upregulated by miR-338, this triggered a mitochondrial dysfunction [98,99], a key cellular sign in AMD [10]. MiR-762 suppressed NADH dehydrogenase subunit 2 (ND2), a core subunit of mitochondrial complex 1, detected in neonatal mouse cardiomyocyte cells. It decreased the ND2 protein level and a knockdown of this miRNA led to increases in ATP levels and complex 1 activity, and decreases in ROS levels and apoptosis in cardiomyocytes [101]. We have previously postulated that decreased ROS and cytoprotection are the key aims in the future therapy of AMD [4].

Three miRNAs, miR-29a, -29b, and -124, suppress the functions of the *MCT1* gene by targeting its 3′-UTR. All of these miRNAs restrain mitochondrial oxidative metabolism [84,85]. The RPE expresses multiple MCT isoforms that are crucial for good retinal health [102]. In contrast to the above-mentioned miRNAs, miR-98 downregulation enhanced oxidative stress and apoptosis, and weakened mitochondrial function and cell viability [90], as observed in an AMD mouse model [27,103]. It is believed that miR-1 targets directly the 3′-UTRs of mitochondrial inner membrane organizing 1 (*MINOS1*) and glycerol-3-phosphate dehydrogenase 2 (*GPD2*) genes. The depletion of these genes evoked disturbances in lipid and carbohydrate metabolism and ETC function, and also triggered mitochondrial damage, manifested by induction of mitophagy. These findings were detected in human breast cancer and melanoma stem cells [72].

In cardiomyocytes isolated from neonatal rats, miR-15b, miR-16, and miR-95 downregulated ATP production by controlling the nuclear gene ADP-ribosylation factor-like 2 (*Arl2*), which is important in the ETC [75]. MiR-494-3p is known to regulate mitochondrial function within RPE cells as the knockdown of this miRNA caused a decrease in mitochondrial function, including a reduction of ATP production and membrane potential. Furthermore, RPE cells treated with rotenone, a selective inhibitor of the mitochondrial respiratory complex I, released extracellular vesicles containing miR-494-3p, reflecting the diminished mitochondrial capacity within these cells. It has been speculated that miR-494-3p could be a useful diagnostic marker for AMD [104]. In contrast, one of the direct targets of miR-494-3p has been found to be PGC-1α, considered as the mitochondrial master regulator in adipocytes [105]. Nonetheless, how this miRNA strengthens mitochondrial capacity in RPE cells needs to be clarified, especially since it might have other targets, and the interactions with these targets could act as a form of compensation to combat the inactivation of PGC-1α. All these observations emphasize that improved mitochondrial energy metabolism and mitophagy might prevent the AMD-related RPE damage [23].

### 5.2. MiRNAs Affecting Additional Mitochondrial Functions Other Than Energy Supply

Next, we discuss the effects of miRNA on other mitochondrial functions, i.e., those unrelated to the energy supply (Table 1). It was reported that miR-19b, a member of a highly conserved 17–92 miRNA cluster (containing miRNAs 17, 18a, 19a/b, 20a, and 92), negatively regulated mitochondrial fusion by suppressing the mitofusin 1 (*MFN1*) gene by targeting its 3′-UTR [76].

The inhibition of miR-23a has protected human RPE cells from H_2_O_2_-induced apoptosis through an upregulation of glutaminase and glutamine uptake. Mechanistically, miR-23a was reported to target the glutaminase 1 (*GLS1*) gene [78]. In addition, this miRNA was claimed to target the 3′-UTR of the manganese superoxide dismutase (*MnSOD*) gene, which is a vital antioxidant enzyme located in the mitochondrial matrix. MnSOD scavenges superoxide and protects cells from ROS. MiR-23a targets the 3′UTR of MnSOD, and thus suppresses its ROS scavenging properties, as shown in mouse cardiomyocytes [79]. In addition, a reduction in MnSOD resulted in AMD-like lesions and was associated with dysregulated energy metabolism, RPE damage, the accumulations of extracellular deposits, thickening of the Bruch’s membrane and the appearance of abnormal blood vessels in the retinal RPE/choroid area [106].

PTEN-induced putative kinase 1 (PINK1) and ubiquitin ligase Parkin (PARK2) are important regulators of the mitochondrial quality control. They induce the disposal of dysfunctional organs, inhibit mitochondrial fragmentation, and reduce ROS production in mitochondria. In a model resembling dAMD, the extent of PINK1/PARKIN-dependent mitophagy was decreased [103]. As detected in human cervical cancer and neuroblastoma cells, miR-27a and b suppress the expression of PINK1 by targeting its gene directly, thus inducing oxidative stress [83]. Finally, there is a report that miR-34a could suppress mitophagy through targeting the *PINK1* gene in human kidney cells and in the mouse brain [87].

MiR-33a and miR-33b suppressed lipid metabolism in the mitochondria of monkey liver cells by reducing the expressions of *CROT* and the hydroxyacyl-CoA dehydrogenase trifunctional multienzyme complex subunit β (*HADHB*). Both of the enzymes coded by these genes are involved in fatty acid oxidation [86]. The decrease in fatty acid oxidation has been related to increased levels of fibrosis [107]. Thus, miR-33a and b might be related to AMD since elevated fibrosis is a characteristic of this disease.

The downregulation of miR-34b and miR-34c has been claimed to inhibit the activity of *PARKIN* and Parkinson’s disease protein 7 gene *PARK7* (DJ-1), an effect mediated by a still unknown mechanism. This finding emerged from assays conducted in post-mortem samples collected from patients suffering from PD. The downregulation of these miRNAs was associated with decreased mitochondrial metabolism and increased oxidative stress and cytotoxicity [89]. Moreover, miR-181a downregulated *PARKIN* in human neuroblastoma cells, leading to a suppression of mitophagy and also to the induction of mitochondrial-mediated apoptosis [94], as observed in the dAMD mouse model [27,103]. In line with this, the downregulation of miR-181a/b strongly protected retinal neurons from cell death since miRNA directly targeted the genes of nuclear respiratory factor 1 (NRF1), COX11, coenzyme Q10 (COQ10; ubiquinone) binding protein COQ10 homologue B (COQ10B), and peroxiredoxin 3 (PRDX3), all of which are important in mitochondrial biogenesis and functioning [95]. An administration of COQ10 has been found to exert beneficial effects on mitochondrial lipid metabolism in early AMD in humans [108].

MiR-101 is known to target the PR domain containing the 16 (*PRDM16*) gene, a member of a protein family involved in cellular proliferation, differentiation, and apoptosis. This targeting was detected initially in silico, but subsequently the effect was detected in human astrocytoma cells. The miRNA interaction decreased the activity of the promoter of *PRDM16*, by targeting methylated histones. This interaction is an example of miRNA-driven transcription regulation. Finally, miR-101 upregulation evoked a disruption of mitochondrial function and the induction of apoptosis, which was reflected in an increased ADP/ATP ratio and elevated caspase-9 level [91]. The mitochondrial-dependent apoptotic signaling pathway in ARPE-19 cells has been observed to be involved in the response to the estrogenically interfering compound, bisphenol A [109].

PGC-1α is recognized as a master regulator of mitochondrial function, and therefore, its abnormal functioning may be important for the occurrence and progression of AMD [25,30]. In human kidney cells, miR-9 upregulated PGC-1α via a so far unknown mechanism, and this miRNA also conferred protection from fibrosis [74]. Furthermore, RPE cells treated with *N*-(4-hydroxyphenyl)-retinamide, which induces elevations in the ROS burden, displayed an increase in the level of miR-9 as a protective process. Thus, miR-9 could be important in the maintenance of the RPE’s functions [110].

MiR-19b-3p, miR-221-3p, and miR-222-3p all have *PPARGC1A* as a common target, as initially evaluated in in silico studies, and their upregulation was associated with a decrease in PGC-1α expression in human atherosclerotic vessel samples, followed by mitochondrial failure and the induction of apoptosis [77]. These miRNAs could thus exert an impact on the degeneration of the RPE via suppression of PGC-1α [27,29]. Additionally, it has been reported that miR-23a-3p could target the mouse *Ppargc1a* gene in mice and suppress mitochondrial function and fatty acid metabolism [80].

MiR-130b-3p has been found to regulate PGC-1α in human placental trophoblastic cells, and an upregulation of this miRNA was linked with a decrease in the amount of mitochondrial transcription factor (TFAM). The extent of ROS induction by 4-hydroxynonenal (4-HNE) was decreased, whereas TFAM expression was increased by exposing the cells to the anti-sense inhibitor of miR-130b-3p [92]. Thus, the manipulation of this micro-RNA could be beneficial in diminishing the level of oxidative stress in RPE. It was reported that 4-HNE levels are elevated in an animal model mimicking the signs of dry AMD [27].

As detected in mouse myoblasts, miR-204 silencing increased PGC-1α mRNA levels, as well as the mitochondrial DNA copy number and citrate synthase activity. Furthermore, this downregulation led to elevations in the autophagy marker microtubule-associated protein 1A/1B light chain 3 (MAP1LC3), and in turn, to the reduced expression of the mitophagy marker FUN14 domain containing 1 (FUNDC1). According to results emerging from in silico studies, this miRNA binds directly to the 3′-UTR of PGC-1α [96]. As autophagy weakening has been proposed to be of importance in AMD [10], these findings might well be relevant with respect to this ocular disease.

### 5.3. Effects of Selected lncRNAs on Mitochondria

To date, eight mitochondrial-encoded lncRNAs (lncND5, lncCyt b, lncND6, MDL1S, MDL1AS, SmtncRNA, ASmtncRNA, and LIPCAR) have been found [111] (Table 2). LncND5, lncCyt b, and lncND6 genes have been detected in human mtDNA, and their coding regions are complementary to the NADH dehydrogenase subunits 5 and 6 (*ND5* and *ND6*) and cytochrome b (*Cyt b*) genes in samples collected from human cervical cancer cells [112]. The functions of the remaining mitochondrial lncRNAs are not known at present [111].

In diabetic mouse models, the lncRNA maternally expressed gene 3 (MEG3) induced mitochondrial fission, whereas MEG3 knockdown suppressed mitochondrial fragmentation and mitochondrial translocation by down-regulating the dynamin-related protein 1 (*Drp1*) gene [113]. In addition, MEG3 acted like a sponge for miR-7, which targets and thus suppresses paired box 6 (Pax6) transcription factor [114]. This protein is claimed to be important in the development of the RPE [115].

There is a report that lncRNA LINC00842 binds to acetylated PGC-1α, and thus prevents its deacetylation by silent information regulator factor 2-related enzyme (SIRT1) in human adenocarcinoma cell lines and adenocarcinoma tissue samples. This leads to a switch of the mitochondrial oxidative process to fatty acid synthesis [116]. It has been demonstrated in a diabetic mouse model that the lncRNA taurine-upregulated gene 1 (TUG1) was bound to an upstream enhancer element of the mouse *Ppargc1a* gene coding PGC-1α protein, leading to an increased PGC-1α expression and improvements in mitochondrial metabolism. Furthermore, downregulation of TUG1 was detected in a glaucoma mouse model, whereas its upregulation relieved the severity of the retinal injury.

**Table 2:** Selected long non-coding RNAs involved in mitochondrial functions or antioxidant response (listed in alphabetical order). Abbreviations are explained in the corresponding text. Arrows (↑ and ↓) indicate up- or down-regulation, respectively.

LncRNA	Target/Mediator	Effects	Model	References
Cyt b ↑	mtDNA (?)	Mitochondrial gene expression regulation (?)	Human cervical cancer cells	[111,112]
FENDRR ↑	*PPARGC1A*/miR-18-5p	Mitochondrial disorder ↓	Human coronary cells	[117]
GAS5 ↑	*Sirt1*/miR-579-3p	Mitochondrial disorder ↓, antioxidant response ↑	Renal injury mouse	[118]
LINC00842 ↑	Acetylated PGC-α	OXPHOS ↓, fatty acid synthesis ↑	Human adeno-carcinoma cells	[116]
MALAT1 ↑	NFE2L2	Antioxidant response ↓	Mouse	[119]
MALAT1 ↑	SMAD 2/3 pathway	EMT ↑	Human RPE cells	[120]
MEG3 ↑	*Drp1*	Mitochondrial fission ↑	Diabetic mouse model	[113]
MEG3 ↑	*MMP-2*	Fibrosis ↑	Mouse cardiac fibroblasts	[121]
MEG3 ↑	*Sirt1*/miR-204	Oxidative stress↓, inflammation ↓	Muller cells of mouse retina	[122]
MEG3 ↑	*NFE2L2*/miR-93	Apoptosis and inflammation ↓	Human RPE cells	[123]
MEG3 ↑	*Pax6*/miR-7	RPE differentiation ↑	Human RPE cells	[114]
ND5 and ND6 ↑	mtDNA (?)	Mitochondrial gene expression regulation (?)	Human cervical carcinoma cells	[111,112]
NRAL ↑	*NFE2L2*/miR-340	Antioxidant response ↑	Human liver carcinoma cells	[124]
PWRN2 ↑	Not known	Cell death ↑, mitochondrial damage ↑	Human RPE cells	[125]
TUG1 ↑	*PPARGC1A*	Mitochondrial function ↑	Diabetic mouse model	[126]
TUG1 ↑	*NFE2L2*	Antioxidant response ↑	Glaucoma mouse model, mouse retinal ganglion cells	[127]
UCA1 ↑	*NFE2L2*/miR-495	Antioxidant response ↑, apoptosis ↓	Rat epilepsy model	[128]

TUG1 upregulation improved antioxidant activity in H_2_O_2_-treated mouse retinal ganglion cells. The mechanism underlying this effect might be the activation of the *NFE2L2* gene by TUG1 [127]. Thus, this lncRNA can exert a dual beneficial effect, promoting both mitochondrial homeostasis and the antioxidant response. In contrast, in a mouse model, *NFE2L2* gene silencing led to the appearance of a dAMD phenotype l [27].

The following two lncRNAs have been shown to induce PGC-1α by inhibiting the functions of specific miRNAs. LncRNA fetal-lethal non-coding developmental regulatory RNA (FENDRR) acted as a sponge for miR-18-5p, a suppressor of PGC-1α expression. As demonstrated in human coronary artery endothelial cells, FENDDR reversed the disturbances in mitochondrial properties induced by oxidized LDL [117]. LncRNA growth arrest-specific transcript 5 (GAS5) and was able to act as a sponge for miR-579-3p. This miRNA downregulated the activity of SIRT1, which consequently led to a downregulation of PGC-1α. Thus, it would seem logical that an upregulation of GAS5 would be able to activate this transcription factor. This has been detected in a sepsis-induced renal injury mouse model [118]. This finding is relevant since PGC-1α downregulation has been associated with the dry AMD phenotype [27,29].

### 5.4. Mitochondrial Actions of Circular Selected Non-Coding RNAs

The circRNAs operating in mitochondria can be divided into endogenous and exogenous forms, but it is poorly known how the latter type reach this organelle. CircRNAs can act as chaperones to facilitate the entry of nuclear-encoded proteins into mitochondria and assist in their folding. It is estimated that hundreds of circRNAs might exist in the mitochondrial genome, but to date, only four mtDNA-derived circRNAs (mecciND1, mecciND5, mc-COX2, and circRNA SCAR) have been functionally annotated [111] (Table 3). However, the biogenesis of these and other mitochondrial circRNAs is not understood, and its mechanism may differ from that occurring in the nucleus as mitochondrial genes do not have any introns [129].

The circular RNAs mecciND1 and mecciND5 are coded by sections of the *ND1* and *ND5* mitochondrial genes, respectively. These circRNAs can serve as chaperones in the proper folding of proteins imported from the cytosol [111]. An upregulation of one mitochondrial-derived circRNA, mc-COX2, has been associated with leukemogenesis and worsening survival. The endogenous suppression of this circRNA impaired mitochondrial functions as it reduced ATP production. In samples gathered from patients with lymphocytic leukemia and also in human cell lines, mc-COX2 was able to inhibit leukemia cell proliferation and induce cell apoptosis [134], but to date, no molecular target for mc-COX2 has been identified. An antisense RNA from the mc-COX2-locus, steatohepatitis-associated circRNA ATP5B regulator (SCAR) was reported to bind to ATP5B, a mitochondrial permeability transition pore regulator. It is known that SCAR is able inhibit both ROS production and fibroblast activation in primary human and mouse liver fibroblasts [139].

CircRNA core-binding factor subunit beta (CBFB) originates from the *CBFB* nuclear gene, coding a transcription factor involved in hematopoiesis. As detected in mouse liver cells and a mouse liver injury model, this circRNA was found to act as a sponge for miR-185, which targets p66Shc, a regulator of mitochondrial ROS production and a mediator of oxidative stress. There is a report that p66Shc is activated in stress conditions, i.e., it oxidizes cytochrome *c* to generate excessive amounts of ROS in the mitochondria [131].

As a summary to Section 5, although the ncRNAs examined here which control mitochondrial function might well be of relevance in the pathology of AMD, this field of epigenetics is still in its early stages, and it may be premature to draw any firm conclusions.

## 6. Non-Coding RNAs in Antioxidant Response Pathway

### 6.1. Overview

In the next sections, the effects of selected ncRNAs in the antioxidant response, namely the NFE2L2/KEAP1 pathway, are discussed (Table 1, Table 2 and Table 3). In addition, some other ncRNAs modulating oxidative stress are mentioned, although this selection is by no means comprehensive. The dysregulation of these ncRNAs in AMD has not been shown directly, but their effects might have an impact on this disease. Theoretically, their manipulation could achieve an upregulation of the antioxidant response, and therefore, they may represent a novel way to reduce the oxidative stress encountered in the RPE and have therapeutic implications.

### 6.2. MiRNAs

In a mouse model of cardiomyocyte hypoxia, miR-24-3p lowered the expression of KEAP1 and it also reduced the amount of apoptosis [81]. Similarly, a downregulation of miR-7 increased KEAP1 expression, in human neuroblastoma cells. It has been found that these miRNAs directly target the 3′-UTR of the gene encoding this sensory protein [73,81]. Nonetheless, with regard to the RPE, miR-7 was reported to exert an opposite effect as it targeted the Pax6 transcription factor [114], which is known to be important in the development of the RPE [115] (See Section 5.3).

In human and rat kidney cell lines, miR-27a directly bound to the NFE2L2 gene and suppressed its expression. It was found that omentin 1, an adipokine compound, reduced miR-27a expression, suppressed oxidative stress and relieved inflammation in kidney cells [82]. While the omentin 1 receptor is still poorly characterized [143], it does seem to be related to the adipokines, and it was observed that the adiponectin receptor 1 (ADIPOR1) variant is associated with advanced AMD [144].

MiR-34a has been shown to increase the progression of PD. The mechanism behind this phenomenon is that in human neuroblastoma cells, it inactivates NFE2L2 and opposes the anti-inflammatory and antineoplastic effects of a natural drug, schisandrin B [88]. Intriguingly, this miRNA has been recently shown to inhibit mitophagy by targeting PINK1 [87]. This finding might be of relevance with regard to AMD therapy that sustains mitophagy via the suppression of miR-34a. In human neuroblastoma cells, it was reported that the upregulations of miR-142, miR-144, and miR-153 could decrease NFE2L2 expression by targeting its 3′-UTR [93]. As a decrease in the amount of NFE2L2 has been detected in AMD [27], the manipulation of miRNAs by inhibiting these miRNAs might be of interest with regard to this disease.

In addition to the KEAP1/NFE2L2 pathway, there are other routes which might have relevance to the progression of AMD. DJ-1 (See Section 5.2), a protein deglycase, modulates the antioxidative response by regulating the expression of superoxide dismutase 1 (SOD1). In addition, it acts as a chaperone for microtubule-associated protein 1 B (MAP1B) to inhibit its aggregation, which in turn would lead to endoplasmic reticulum stress-induced apoptosis. One of the miRNAs, miR-494, can downregulate DJ-1. As studied in mouse adipocyte and neuroblastoma cells, an overexpression of miR-494 was shown to increase oxidative stress [100]. In post-mortem samples from PD patients, miR-34b and miR-34c downregulation led to a suppression of DJ-1 [89], as already discussed in Section 5.2.

### 6.3. LncRNAs

LncRNA TUG1, originating from the *TUG1* gene (Section 5.3), has been shown to be downregulated in a glaucoma mice model subjected to an ischemic reperfusion and in a H_2_O_2_-treated mouse retinal ganglion cell line. The ROS scavenger chlorogenic acid has been found to upregulate lncRNA TUG1. As predicted by a bioinformatics analysis, it was speculated that this lncRNA is able to upregulate the *NFE2L2* gene. Conversely, inhibition of lncRNA TUG1 resulted in the degradation of the NFE2L2 protein. A direct interaction between lncRNA TUG1 and the NFE2L2 protein has been reported. Since it is expressed in the retina, this lncRNA is of special interest with regard to the oxidative stress encountered in AMD [127].

The metastasis-associated lung adenocarcinoma transcript 1 (MALAT1) lncRNA overexpression has been associated with many diseases, as well as with normal development and organism viability [145]. For example, it inhibits the transcriptional activity of the NFE2L2 protein. MALAT1-null mice exhibited an upregulation of the genes controlled by NFE2L2 in conditions of oxidative stress. In addition, the insulin response was increased in these mice after their exposure to ROS [119]. With regard to AMD, MALAT1 has been shown to induce transforming growth factor beta (TGF-β)-induced EMT in ARPE-19 cells, and in addition, it might be able to interact with the proteins of the signal transducer SMAD2/3 pathway, which is important in EMT [120]. These findings emphasize the importance of lncRNAs in the regulation of NFE2L2 in oxidative stress. Several lncRNAs upregulate NFE2L2 by acting as sponges for one of its inhibitor miRNAs. For example, NFE2L2 regulation-associated lncRNA (NRAL), which sponges miR-340, has been detected in human liver carcinoma cell lines and tissue samples [124]. Urothelial carcinoma associated 1 (UCA1) is another example; this lncRNA was found in a rat epilepsy model, where it was reported to act as a sponge for miR-495. In addition to increasing the antioxidant response, UCA1 decreased apoptosis and inhibited neuronal injuries [128], and furthermore, lncRNA GAS5 increased the level of NFE2L2 by upregulating SIRT1 via a sponging of miR-579-3p [118].

LncRNA MEG3 was discussed above (Section 5.3) as an upregulator of mitochondrial fission. It was also demonstrated to be a promoter of fibrosis by increasing the expression of matrix metalloproteinase 2 (MMP-2) in mouse cardiac fibroblasts [121]. Conversely, it seemed to be involved in a decrease in oxidative stress in the Muller cells of the mouse retina. The pineal hormone, melatonin, was able to upregulate the expression of MEG3. This lncRNA acted as a sponge for miR-204, an inhibitor of SIRT1, which led to its upregulation and this was followed by increases in the deacetylation of forkhead box O1 (FOXO1) and nuclear factor kappa B (NF-κB) subunit p65, both of which are known to contribute to the alleviation of oxidative stress and inflammation [122]. Furthermore, activation of FOXO1 signaling is associated with increased autophagy [146], and NF-κB regulated autophagy [147]. In addition, there is a report that MEG3 is able to inhibit apoptosis and inflammation in RPE cells by sponging miR-93, which in turn targets *NFE2L2* [123].

The Prader–Willi region non-protein coding RNA 2 (PWRN2) has been shown to be upregulated in human ARPE-19 cells following exposures to H_2_O_2_, tert-butylhydroperoxide, or UVB. The suppression of this lncRNA was revealed to alleviate cell death, apoptosis and mitochondrial injuries in conditions of oxidative stress [125]. These data suggest that PWRN2 could well have an influence in regard to AMD.

### 6.4. CircRNAs

In human liver cells, the level of one circRNA, hsa_circ_0005915, was upregulated by the oxidative stress evoked by *N*,*N*-dimethylformamide and subsequently, it promoted the ubiquitination and degradation of NFE2L2, which was followed by elevated ROS production [132]. It is known that circKEAP1 controls *KEAP1* by acting as a sponge for its inhibitor miR-141-3p. This circRNA is coded by the second exon of the human *KEAP1* gene, and a suppression of circKEAP1 triggers the release of miR-141-3p. As well as activating the NFE2L2 antioxidant response, this miRNA was demonstrated to inhibit cell proliferation and migration via a mechanism elucidated in lung adenocarcinoma primary cells and some other adenocarcinoma cell lines [133].

Circular RNA arginine-glutamic acid dipeptide repeats (circ_RERE) were able to stimulate H_2_O_2_-induced oxidative stress in human nucleus pulposus (NP) cells from the inner core of the vertebral disc by promoting apoptosis and autophagy. In addition, this circRNA induced the expression of galectin-3, a protein involved in various pathophysiological states, including fibrosis. Mechanistically, circ_RERE was shown to act as a sponge for miR-299, a regulator of galectin-3 expression [137,138], which makes this circRNA possibly relevant in AMD since fibrosis occurs in the later phases of this disease [148]. CircPRKCI originates from two exons of the protein kinase C iota (*PRKCΙ*) gene; it is downregulated in H_2_O_2_-treated human neuroblastoma SH-SY5Y cells. As this circRNA was a sponge for miR-545 and miR-589, these two miRNAs were released and accumulated after cells were exposed to H_2_O_2_. This led to decreased expression of their target, E2F transcription factor 7 (E2F7), and mediated an increase in the severity of the cell injury [136].

The origin of circSLC8A1 is in one exon of the solute carrier family 8 member 1 (*SLC8A1*) gene, and it has many miRNA binding sites. The induction of chemical oxidative stress in human neuroblastoma cells increased the expression of this circRNA. Concordantly, simvastatin, an antioxidant modifier, decreased the expression of circSLC8A1. With regard to the miRNAs, circSLC8A1 has seven binding sites for miR-128, and thus it can act as an efficient sponge for this miRNA [140]. Since miR-128 inactivated the axis inhibition protein 1 (*AXIN1*) gene, it helped to protect neurons from apoptosis; conversely, when the amount of circSLC8A1 was increased, this protective effect was weakened due to miR-128 sponging [141].

The amount of circAKT3 RNA, which is derived from the protein kinase B gamma gene, is increased in ischemic conditions; this phenomenon has been detected in a rat renal ischemic model as well as in human and rat renal cells. It was reported that circAKT3 RNA acted as a sponge for miR-144, a repressor of the β-catenin/WNT (Wingless and Int-1) signaling, which promotes apoptosis and the EMT process in the cell, a phenomenon connected to fibrosis [130]. The plasma membrane Na^+^/Ca^2+^ exchanger gene-derived circNCX1 was increased in the presence of ROS, and it promoted apoptosis in rat myocardial cells and in cardiac cells from a mouse ischemia model. The mechanism of action seemed to be its ability to act as a sponge for miR-133-3p. This micro-RNA suppressed the expression of pro-apoptotic gene cell death-inducing protein 1 (*CDIP1*) [135].

CircRNA sperm antigen with calponin homology and coiled-coil domains 1 gene (circ-SPECC) acted as a sponge for miR-33a, which in turn directly controlled the transforming growth factor beta 2 (*TGFβ2*) gene, a factor involved in regulating cell growth and division. Circ-SPECC1 was reported to be downregulated in H_2_O_2_-treated human hepatocarcinoma cells, which led to a promotion of apoptosis and inhibition of their proliferation. In addition, it has been demonstrated that upregulation of miR-33a evoked a suppression of autophagy [142].

## 7. Non-Coding RNAs as Therapeutic Targets

### 7.1. General Aspects

MiRNAs have been the most intensively studied species in the current and prospective therapeutic applications of the ncRNAs. The applications include either miRNA mimics or (agomirs), which resemble the original miRNA sequence and mimic its action, or antagomirs, which contain the complementary sequence of the miRNA in question. The latter can pair with the endogenous miRNA and thus block its action. MiRNA mimics have been chemically modified to prevent their degradation and increase their RNA and protein binding properties [149,150]. The difficulty in therapeutic applications involving miRNAs, in addition to the route of their delivery, is the fact that these RNA species have various targets [44,151].

In comparison to many other tissues, the eye provides unique properties for the delivery of miRNA mimics or antagomirs. NcRNAs packed into suitable vehicles can be directly injected into the vitreous humour; from there the RNA could be transferred to the retina and reach the RPE. The capacity of the cells of the RPE to phagocytosize external material is an important characteristic in this process. Therefore, the vehicles could be double-membrane covered extracellular vesicles, sized ca. 30–100 nm, which could be produced in donor cells, and would pack the introduced miRNAs into vesicles; these packages would then be released from these cells by exocytosis, and extracted for use [4]. Another approach could be the miRNA transfer by 1–100-nm sized artificial nanoparticles [150].

While there are few reported pathogenic connections between lncRNAs, and especially circRNAs, and AMD, as seen here (Section 5 and Section 6), more data exist on miRNAs. However, many ncRNAs have effects on mitochondrial function and the antioxidant stress response, both of which are important in the pathology of AMD. In addition, the manipulation of the ncRNAs mentioned here could be useful in other diseases associated with mitochondrial malfunctions or defects in the antioxidant response.

### 7.2. MiRNAs

There are several reports that the downregulation of miR-26a/b, miR-181c, miR-210, and miR-762 can induce OXPHOS [65,67,69]. As miR-181a and b target several of the genes important in mitochondrial function, such as NRF1 and those involved in mitophagy [94,95], their suppression could well be beneficial in the maintenance of mitochondrial function.

A downregulation of miR-181a and b would be able to protect retinal neurons from death as these miRNAs directly target the genes of nuclear respiratory factor 1 (NRF1), COX11, coenzyme Q (ubiquinone) binding protein COQ10 homologue B (COQ10B), and peroxiredoxin 3 (PRDX3), all of which are important in mitochondrial biogenesis and functioning [95]. With respect to PGC-1α activation, one can speculate that the upregulation of miR-9 [74], and downregulation of miR-204 [96], as well as that of miR-32a-3p (Wang et al., 2022) [80], might have a beneficial effect in the prevention of the degeneration of the RPE [29,103].

It has been reported that an upregulation of miR-24-3p KEAP1 was able to increase the activity of NFE2L2 [81]. The group of miRNAs 142, 144, and 153 are known to target NFE2L2 directly, and their downregulation is strengthened in times of antioxidant signaling [93]. As an example of how an increase in the level of a miRNA could be used as a potential therapy, it was claimed that the upregulation of miR-98, a modulator of Notch signaling, might reduce oxidative stress, enhance mitochondrial function and improve cell viability [90]. In addition, the expression levels of two of the ROS scavengers, i.e., MnSOD and glutaminase 1, were reported to be induced by miR-23a inhibition [78,79].

### 7.3. LncRNAs

To date, although no lncRNA-targeted therapy has been entered into clinical trials, their use as biomarkers has been explored, especially in cancers. For example, the level of long non-coding RNA-activated by transforming growth factor β (ATB) was increased by 5–10-fold in glioma patients, while that of a metastatic prostate cancer-related lncRNA PCAT18 was reported to be elevated by 8.8–11-fold in prostate cancer cells [152].

When one considers the suppression of lncRNA expression, it would be possible to exploit an RNA interference technique. In this approach, short interfering RNAs (siRNAs) have the potential for suppressing lncRNAs. Natural antisense transcripts and CRISPR (clustered regularly-interspaced short palindromic repeats) methodology could be applied to target against lncRNA genes as these techniques have been employed to achieve a downregulation of these ncRNAs [52]. If the aim was to increase the expression of lncRNAs, it might be possible for them to be cloned into lentiviral cassettes and transduced into cells. Lentiviruses produce single stranded RNAs, and can carry up to 10-kb inserts [153]. Another application would be adenoviral-mediated delivery [150], and novel transposon vector techniques are emerging all the time [154].

Suppression of lncRNA MALAT1 increased the phenomenon of EMT [120]. This has been putatively linked with wAMD and reductions in the antioxidant response [119]. Similarly, a decrease in lncRNA PWRN2 might be able to relieve mitochondrial damage and RPE cell death and thus be an effective therapeutic approach in AMD [125]. Upregulation of PGC-1α-activating lncRNAs FENDRR [117] and TUG1 [126] might be beneficial as this would be one way to support optimal mitochondrial functioning. If the goal was to strengthen NFE2L2-signaling, then increases in the amounts of lncRNAs NRAL [124], TUG1 [127] and UCA1 [128] might be considered.

Downregulation of the degradation useful lncRNAs by small molecules could be a method to increase their levels. For example, the lncRNA, GAS5, targets miR-579-3p, which leads to SIRT1 activation. Consequently, PGC-1α and NFE2L2 would become activated, which would lead to a reduction of mitochondrial damage [118]. Furthermore, there is a report that a small molecule NP-C86 was able to stabilize GAS5 by preventing its degradation by regulator of nonsense transcripts 1 protein, a post-splicing factor participating in the junctioning of exons [155].

### 7.4. Circular ncRNAs

As circRNAs are generally more stable than other types of RNA, they have attracted interest in the therapy of cancer and some other diseases [156]. An overexpression of endogenous as well as the introduction of engineered or synthetic circRNAs or circRNA mimics, could be used in these applications. Adeno-associated virus cassettes have been exploited as a means to introduce circRNAs into therapy [60]. CircFndc3b is a good example of the effective exploitation of circRNA as it has been shown to enhance cardiac function by regulating VEGF signaling [157].

The upregulation of circ-SPECC1 could represent a possible therapeutic target since this species is downregulated in oxidative stress, and not only does it promote apoptosis, but it also has a capacity to promote autophagy [142]. It has been speculated that inhibition of circ_0005915 might promote the stimulation of an antioxidant response [132]. Another possibility would involve the silencing of AKT3 as this could lead to suppression of EMT and apoptosis [130], and this might also apply to NCX1 in the downregulation of apoptosis [135]. It would be interesting to examine if downregulation of the circRNA KEAP1 [133] which acts as a sponge for miR-144, a *KEAP1* gene inhibitor, would be able to activate the NFE2L2-mediated antioxidant response.

## 8. Conclusions

Mitochondria and the signaling which occurs as a response to oxidative stress are putative therapeutic targets against AMD. The control of the expressions of genes by epigenetic means, such as manipulating the amounts of specific ncRNAs, is a promising future prospect. Many of the ncRNAs discussed here are connected to autophagy, and a weakening of this process is encountered in AMD. Autophagy is connected to mitochondrial function and the signaling to trigger an antioxidant response via PGC1α and NFE2L2, respectively, as discussed in the recent reviews by Hyttinen and others [4,34].

The initial problem in ncRNA therapeutics is their specificity. As these molecules can target many genes, undesired effects must be prevented. The next obstacle is the delivery of ncRNAs, and efficient transfer vehicles will be needed for targeting them to the correct organ and eventually to the cell type requiring treatment. Concerning RNA molecules, further problems arise from their general instability, especially when they are “naked” and chemically unmodified. Finally, there are tolerability issues due to the recognition of the delivered ncRNAs by pathogen-associated molecular pattern receptors (e.g., Toll-like receptors) in the cell, which would lead to adverse immunological outcomes [52].

As already addressed, much progress is taking place in the field of ncRNA research. This is especially true with regard to the lncRNAs and circRNAs because their actions are still largely unexplored. It seems likely that many important regulatory mechanisms mediated by ncRNAs will appear in the future, and these may well be novel targets for future therapeutic applications as well as diagnostic markers for many diseases, such as the regulation of mitochondrial function and the oxidative stress response. Thus, ncRNAs might be useful in developing novel therapies against AMD. Future transcriptomics studies, including improvements in single-cell qPCR methodology, deep sequencing (i.e., sequencing of the genomic region of concern several times for detecting rare sequences), spatial-dependent sequencing, and more generally, progress in the bioinformatics methodologies [158,159] will no doubt increase our knowledge of the complex topic of non-coding RNAs.

In the future, it may be possible to devise a truly personally tailored therapy against AMD. This would involve assessing the patient’s ncRNA profile, and then an individual therapy would be designed according to this data. Although there are still many questions to be answered and obstacles to be overcome in this field, new promising innovative developments will undoubtedly emerge in the future. In addition, these solutions could be exploited in the diagnostics and therapy of other degenerative diseases, which are also increasing in the ageing populations in a similar manner to AMD.

## Figures and Tables

**Table 3:** Selected circular non-coding RNAs related with mitochondrial functions or antioxidant response (listed in alphabetical order). The abbreviations are described in the corresponding text. Arrows (↑ and ↓) indicate up- or down-regulation, respectively.

CircRNA	Target/Mediator	Effects	Models	References
AKT3 ↑	β-catenin-Wnt signaling/miR-144	Apoptosis ↑, EMT ↑	Rat renal ischemic model	[130]
CBFB ↑	*p66Shc*/miR-185	Mitochondrial ROS ↑	Mouse liver injury model, and mouse liver cells	[131]
circ_0005915 ↑	NFE2L2 pathway	Antioxidant response ↓	Human liver cells	[132]
KEAP1 ↑	*KEAP1*/miR-141-3p	Antioxidant response ↓	Human lung adenocarcinoma samples	[133]
mc-COX2 ↓	?	ATP production ↓	Leukemia samples, leukemia cells	[134]
mecciND1 and mecciND5 ↑	Mitochondrial proteins	Protein import ↑, chaperone function ↑	Human cervical cancer cells	[111]
NCX1 ↑	*CDIP1*/miR-133-3p	Apoptosis ↑	Rat myocardial cells, and mouse ischemia model	[135]
PRKCI ↓	*E2F7*/miR-545 and miR-589	Neuronal cell injury ↑	Human neuroblastoma cells	[136]
RERE ↑	*Galectin-3*/miR-299	Apoptosis ↑, fibrosis ↑	Human nucleus pulposus cells	[137,138]
SCAR ↑	*ATP5B*	ROS production ↓, fibroblast activation ↓	Human and mouse fibroblasts	[139]
SLC8A1 ↑	*AXIN1*/miR-128	Apoptosis ↑	Human neuroblastoma cells	[140,141]
SPECC1 ↓	*TGFβ2*/miR-33a	Apoptosis ↑, proliferation ↓, autophagy ↓	Human hepatocarcinoma cells	[142]

## Data Availability

All data is retrieved from public literature databases (PubMed, National Library of Medicine).

## Abbreviations

AD	Alzheimer’s disease
AKT	protein kinase B
AMD	age-related macular degeneration
ASO	antisense oligonucleotide
circRNA	circular RNA
COQ10	coenzyme Q10; ubiquinone
COX	cytochrome c oxidase subunit
CREB	cAMP response element binding protein
CROT	carnitine-*O*-acetyltransferase
dAMD	dry AMD
EMT	epithelial-to-endosomal transition
ETC	electron transport chain
KEAP1	Kelch-like ECH-associated protein 1
LNA	locked nucleic acid
lncRNA	long non-coding RNA
miRNA	microRNA
MnSOD	manganese superoxide dismutase
mtDNA	mitochondrial DNA
ncRNA	non-coding RNA
ND	(mitochondrial) NADH dehydrogenase subunit
NFE2L2	nuclear factor erythroid 2-related factor 2; NFE2 like bZIP transcription factor 2
NOX	NADPH oxidase
NRF	nuclear respiratory factor
nt	nucleotide
OXPHOS	oxidative phosphorylation
PD	Parkinson’s disease
PGC-1α	peroxisome proliferator-activated receptor gamma coactivator-1 alpha
PINK1	PTEN-induced putative kinase 1
*PPARGC1A*	gene coding PGC-1α
ROS	reactive oxygen species
RPE	retinal pigment epithelium
SIRT1	silent information regulator factor 2-related enzyme
TFAM	mitochondrial transcription factor
TGF	transforming growth factor
UTR	untranslated region
VEGF	vascular endothelial growth factor
wAMD	wet AMD
